# Editorial: Photo/electrocatalysis for energy storage and conversion

**DOI:** 10.3389/fchem.2023.1173756

**Published:** 2023-03-23

**Authors:** Fei Pan, Zhuo Wang, Menglan Lv, Bin Zhang

**Affiliations:** ^1^ School of Chemistry and Chemical Engineering, Guizhou University, Guiyang, China; ^2^ School of Chemical Engineering, Guizhou Institute of Technology, Guiyang, China

**Keywords:** polymer acceptors, polymerized small-molecule acceptors, Y-series small molecule acceptors, high efficiency, all-polymer solar cells

Polymer solar cells (PSCs) have drawn great attention as a hopeful renewable energy sources technology due to their advantages in mechanical flexibility, light weight and large-scale roll-to-roll fabrication. Recently, the considerable achievement of PSCs has benefited from the development of novel photovoltaic materials and the modulation of active layer morphology. Up to now, the power conversion efficiency (PCE) of PSCs using p-type polymer as the donor and n-type small molecule as the acceptor has exceeded 19%. Among them, the all-PSCs are considered as one of the most promising candidates for commercial applications ascribing to the higher thermal stability and mechanical flexibility. With tremendous effort being devoted to the design and synthesis of polymer acceptor materials, including perylene diimide (PDI), nanphthalene diimide (NDI), B ← N-bridged bipyridine polymer, and polymerized small molecule acceptors (PSMAs), the photovoltaic performance has achieved significant improvement with PCE of > 18%. Compared to the PDI-, NDI- and B ← N-typed polymer acceptors, the PSMAs is caught much more attention resulting from their wider absorption and stronger absorption coefficients.

**FIGURE 1:**
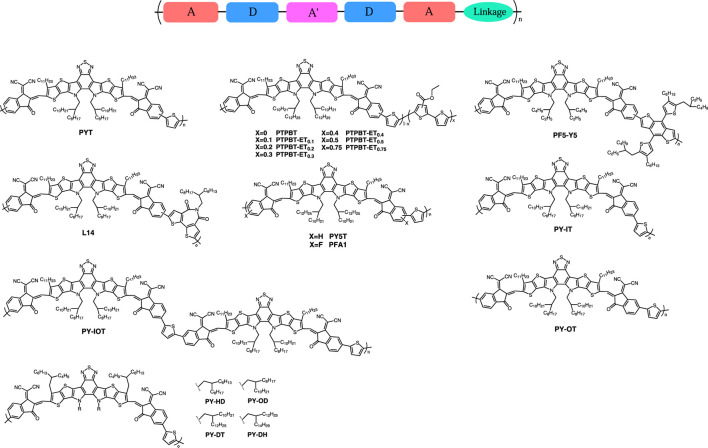
The chemical structures of typical Y-series PMSAs.

In order to further improve the PCE of all-PSCs, it is crucial to synthesize high-performance polymer acceptors and finely adjust the active layer morphology. Due to the great success of Y-series SMAs in PSCs, a widely used method for synthesizing polymer acceptors is the polymerization of Y-series SMAs (Figure 1). Wang et al. (2020) reported a narrow bandgap PMSA of PYT using Y5-C20 as the building block and thiophene as the bridging unit. The effects of different molecular weights on the optical and electrical properties of the PYT, and the morphology of the active layer were also investigated in detail. The results showed that the medium molecular weight for PYT exhibited suitable miscibility with PM6, which was favorable for obtaining more balanced carrier mobility, stronger intermolecular aggregations, more ordered features, higher charge-transport ability and less energy loss, resulting in the higher photovoltaic performance of 13.44%, compared to the ones of low and high molecular weights.

Moreover, when using the random copolymerization of three different functional units on the molecular backbone, the opto-electrical properties such as energy level and absorption spectrum of the resulting polymer could be easily tuned by changing the molar ratio of different moieties. Based on this strategy, Du et al. (2020) synthesized a series of terpolymer PMSAs PTPBT-ET_x_s by randomly copolymerizing 3-ethylesterthiophene (ET) with A-DA'D-A typed SMA unit (TPBT-Br) and thiophene-bridged units. It was found that the lowest unoccupied molecular orbital (LUMO) of the obtained PMSA gradually shifted upward with increasing ET content, which favored to obtain higher *V*_oc_, mainly due to the weak electron-pulling property and the higher frontier orbital energy level of the ET unit. Notably, it suggested that the ester group on the ET may cause non-covalent intra- and intermolecular S•••O interaction, leading to the formation of more planar molecular backbone and thus the preferential face-on orientation of the molecular arrangement. Matching with PBDB-T as donor to prepare all-PSCs, the PCE of PTPBT-ET_0.3_ as the acceptor is 12.52%.

In addition to the simple thiophene-like π-bridges, other donor units have been reported to be introduced as bridging units into the Y-series PMSAs. Fan et al. (2020) reported a PMSA PF5-Y5 by copolymerizing the classically efficient donor unit thienyl-benzodithiophene (BDT-T) as a bridging unit with Y5. Compared to the corresponding SMA Y5, PF5-Y5 showed a redshift of 25 nm in solution due to the extension of the conjugated backbone, and a blue shift of 10 nm in film attributing to the introduction of the non-conjugated alkyl side chain on BDT-T and thus inhibiting the excessive aggregation and phase separation. Therefore, the PBDB-T:PF5-Y5-based all-PSC presented higher PCE of 14.45% with smaller non-radiative loss of 0.24 V and larger open-circuit voltage of 0.95 V, compared to the PBDB-T:Y5-based PSC.

Furthermore, the strategy of using electron-deficient units to copolymerize with SMAs to construct A-A-type polymer acceptors is believed to improve electron mobility and facilitate electron transport potentially. To obtain both stronger light absorption and higher electron transport properties for polymer acceptor, Sun et al. (2020) reported a novel PMSA (L14) by introducing distannylated bithiophene imide derivatives (BTI-Tin) as the electron-deficient building block and Y5 as core into the polymer backbone. The L14 showed a broad and strong absorption in the 600–900 nm band with a higher absorption coefficient, compared to thiophene-bridged counterpart L11 (or named PYT). The more balanced and higher carrier mobility, denser π-π stacking, and more ordered and predominant face-on molecular arrangement in PM6:L14 film can facilitate the charge transport in the vertical direction compared to PM6:L11 system. The PCE of all-PSCs based on PM6:L14 was 14.3%, which was higher than that of PM6:L11 with 11.1%.

Additionally, the introduction of strongly electronegative fluorine (F) atoms could significantly decrease the molecular frontier orbital energy level and enhance inter-/intra-molecular interactions, which is an effective way to modulate the energy level and molecular stacking in PSCs. Peng et al. (2020) introduced F atoms into the structure of polymer acceptor, the fluorinated polymer PFA1 possessed a slightly down-shifted energy level, narrower bandgap, higher absorption coefficient, and more balanced charge mobility compared to the non-fluorinated counterpart PY5T. In addition, PFA1 showed better miscibility and ordered stacking morphology with the polymer donor, resulting in the better PCE of 15.11%.

At last, a mixture of two isomers (brominated 1,1-dicyanomethylene-3-indanone, IC-Br) that are difficult to separate each other, is now widely used as the terminal for the polymerization of SMAs. This unit not only causes batch differences that are difficult to reproduce, but also reduces the ordered stacking of morphologies and efficient charge transport, resulting in lower photovoltaic performance than the SMAs based PSCs. On the basis of this consideration, Luo et al. (2020) developed two structurally determined pure IC-Br-based monomers by recrystallization in different solvents and used them to polymerize with Y5-liked SMAs for the synthesis of PMSAs. Two PMSAs with defined polymerization (PY-IT and PY-OT) and one with random polymerization (PY-IOT) were synthesized to study the optoelectronic and photovoltaic properties of polymer acceptors. Compared to the PY-OT and PY-IOT, the PY-IT showed smaller bandgap, larger absorption coefficient, lower LUMO energy level, larger and more balanced charge mobility and more suitable phase separation morphology. As a result, all-PSCs based on PM6:PY-IT obtained a PCE of 15.05%, which was significantly higher than PM6:PY-OT of 10.04% and PM6:PY-IOT of 12.12%. The authors noted that the development of polymer acceptors with defined and optimized structure could facilitate the acquisition of highly efficient all-PSCs. Similarly, Li et al. (2022) reported a series of PSMAs with defined polymerization site using the core of SMA L8-BO as the building block. By tuning the optical and electrical properties of the polymer through the adjustment of the alkyl side chain length, the PM6:PY-DT-based all-PSCs obtained a high PCE of 16.76% with a low non-radiative loss of 0.18 V.
